# Estimates and predictors of HIV viral non‐suppression in South African adults on antiretroviral treatment

**DOI:** 10.1002/jia2.70076

**Published:** 2026-01-19

**Authors:** Haroon Moolla, Reshma Kassanjee, Jonathan Euvrard, Gary Maartens, Hans W. Prozesky, Matthew P. Fox, Catherine Orrell, Geoffrey Fatti, Frank Tanser, Gilles Wandeler, Mary‐Ann Davies, Renee de Waal, Patience Nyakato, Leigh F. Johnson, the International epidemiology Databases to Evaluate AIDS Southern Africa (IeDEA‐SA) Collaboration

**Affiliations:** ^1^ Centre for Integrated Data and Epidemiological Research School of Public Health University of Cape Town Cape Town South Africa; ^2^ Division of Clinical Pharmacology Department of Medicine University of Cape Town Cape Town South Africa; ^3^ Division of Infectious Diseases Department of Medicine Stellenbosch University and Tygerberg Hospital Cape Town South Africa; ^4^ Departments of Epidemiology and Global Health Boston University School of Public Health Boston University Boston Massachusetts USA; ^5^ Health Economics and Epidemiology Research Office Faculty of Health Sciences University of the Witwatersrand Johannesburg South Africa; ^6^ Desmond Tutu Health Foundation Cape Town South Africa; ^7^ Division of Epidemiology and Biostatistics Department of Global Health Faculty of Medicine and Health Sciences Stellenbosch University Cape Town South Africa; ^8^ Africa Health Research Institute (AHRI) KwaZulu‐Natal South Africa; ^9^ South African Centre for Epidemiological Modelling and Analysis (SACEMA) Centre for Epidemic Response and Innovation Stellenbosch University Stellenbosch South Africa; ^10^ School of Nursing and Public Health University of KwaZulu‐Natal Durban South Africa; ^11^ Department of Infectious Diseases Bern University Hospital University of Bern Bern Switzerland

**Keywords:** HIV, antiretroviral therapy, dolutegravir, viral load, interrupted treatment, longitudinal study

## Abstract

**Introduction:**

Viral suppression estimates are essential for monitoring the performance of HIV programmes. South Africa introduced dolutegravir (DTG)‐based antiretroviral therapy (ART) in 2019. We sought to generate updated estimates of viral suppression in South African adults on ART and investigate predictors of viral non‐suppression.

**Methods:**

This retrospective cohort study used data from seven South African cohorts participating in the International epidemiology Databases to Evaluate AIDS collaboration. Three main analyses were performed using a viral suppression threshold of 1000 HIV RNA copies/ml. In the first analysis, we fitted a logistic regression model using the full data from the study period (2005−2023). Then, in two causal analyses, we used logistic regression with inverse probability weighting to assess the effects of starting ART on DTG‐based regimens (as opposed to starting on non‐DTG‐based ART) and switching to DTG while virally suppressed (compared to remaining on non‐DTG‐based ART). In sensitivity analyses, we reduced the suppression threshold to 400 copies/ml and excluded those with missing baseline CD4+ cell count measurements.

**Results:**

There were 380,720 participants contributing 2,090,912 person‐years of observation. Most participants were female (64.7%), and the median age at ART initiation was 35.0 years (interquartile range 28.9−42.3). Viral suppression increased over time, reaching 95.9% in 2023. Twenty‐one percent of participants either started ART on DTG‐based regimens (7.1%) or switched to DTG‐based regimens from a virally suppressed state (14.0%). DTG‐based ART was protective against viral non‐suppression in both causal models, with adjusted odds ratios of 0.54 (95% confidence interval [CI] 0.48−0.61) and 0.36 (95% CI 0.32−0.39) for those initiating ART on DTG and those switching to DTG, respectively. A history of ART interruption was strongly associated with viral non‐suppression, with adjusted odds ratios ranging from 2.49 to 4.55. The odds of non‐suppression decreased with increasing age, increasing duration on ART and increasing baseline CD4+ cell count. Results were consistent across sensitivity analyses.

**Conclusions:**

DTG‐based regimens improve viral suppression among both ART‐naïve individuals and those transitioning while suppressed. ART interruptions pose a risk to the sustained success of ART programmes and may further impede efforts to recover from the impacts of recent funding cuts.

## 1 INTRODUCTION

People living with HIV (PLHIV) who are virally suppressed on antiretroviral therapy (ART) experience faster increases in CD4+ cell counts and, therefore, lower mortality [1, 2], lower rates of progression to AIDS [3] and lower rates of non‐AIDS‐defining cancers [4, 5]. Population‐level viraemia is strongly associated with higher HIV incidence rates and is a crucial determinant of epidemic control [6, 7, 8, 9, 10]. The inclusion of viral suppression as the third of the UNAIDS 95‐95‐95 targets highlights its importance in HIV programme monitoring.

South Africa has the largest ART programme in the world [11], with routine viral load monitoring included in ART guidelines from the start of the national ART programme in 2004 [12]. In 2019, Pillay et al. [13] estimated the proportion of adults with viral suppression in South African cohorts participating in the International epidemiology Databases to Evaluate AIDS (IeDEA) collaboration. Viral suppression was found to be on a gradual upward trend, reaching 92.2% in 2018 (using a threshold of 1000 copies/ml).

The introduction of dolutegravir (DTG) in November 2019 may have altered this proportion. DTG‐based ART regimens, which are more efficacious and robust than non‐nucleoside reverse transcriptase inhibitor‐based regimens [14], are expected to have improved viral suppression. However, observational studies in low‐ and middle‐income countries have found a wide range of effect sizes [15, 16, 17]. The impact of the COVID‐19 pandemic is also uncertain. In South Africa, HIV services were disrupted early during the pandemic, with ART initiation and viral load testing declining by 28% [18] and 22% [19], respectively. However, an analysis of data from a private sector cohort found that viral suppression increased during the pandemic [20]. Concurrently, many countries, including South Africa, have progressively expanded the implementation of longer ART dispensing intervals and reduced frequency of clinical visits [21, 22]. A 2022 review of outcomes associated with reduced frequency of visits compared to standard visit schedules found inconsistent results for viral suppression, largely due to the high proportion of missing viral load measurements [23].

Considering these recent events, the primary objective of this study is to generate updated estimates of viral suppression in adults on ART. The secondary objective is to estimate the effects of predictor variables on viral non‐suppression. Here, we aimed to answer two questions related to DTG use. First, among those newly initiating ART, what is the effect of starting DTG‐based ART compared to starting non‐DTG‐based ART? Second, among those on non‐DTG‐based regimens with viral suppression, what is the effect of switching to DTG‐based ART?

## 2 METHODS

### 2.1 Study design and setting

2.1

This was a retrospective cohort study using data from seven South African cohorts of the IeDEA‐SA Collaboration (Aid for AIDS, Gugulethu, Hlabisa, Khayelitsha, Khethimpilo, Themba Lethu and Tygerberg). This collaboration has been described previously [24]. Briefly, demographic data, dates of clinic visits, details of ART, and dates and results of laboratory tests are collected prospectively.

### 2.2 Study population

2.2

The study population included all adults (15 years or older) living with HIV who initiated ART for the first time between January 2005 and December 2023. For each cohort, we only included data from the first year in which viral load testing was performed for at least 40% of participants. This was done to ensure that there were sufficient data for imputing missing viral load measurements. We excluded those with viral loads less than 400 copies/ml at ART initiation, as these participants were likely not ART‐naïve. Of the seven cohorts, four did not contribute data in later years, as these later data can only be analysed within a separate secure data environment and could not be included in a combined analysis alongside the rest of the data. The final year of data contributed for these four cohorts were 2020 for Tygerberg, 2021 for Gugulethu and 2022 for Khayelitsha and Themba Lethu.

### 2.3 Variables

2.3

For each participant, their baseline was the date of first ART initiation. Baseline CD4+ cell counts were taken as the last measurement in the window from 6 months before to 7 days after first ART initiation. Missing baseline CD4+ cell count measurements were imputed using a regression model that included sex, baseline age, year of ART initiation, cohort and initial ART regimen (DTG vs. non‐DTG) as explanatory variables.

We used two indicator variables to classify DTG use: participants who started DTG‐based regimens within 30 days of ART initiation were classified as having “started on DTG,” whereas those who switched from non‐DTG‐based regimens to DTG‐based regimens while virally suppressed were classified as having “switched to DTG.” For the latter group, their measurements prior to switching were included in the non‐DTG group. The condition of being virally suppressed prior to switching aligns with the South African national ART guidelines, which advised that people only be switched to DTG if they had viral suppression within the preceding 6 months. To ensure that participants who transition to second‐ or third‐line ART are all analysed equivalently, after starting or switching to DTG from a suppressed state, all their subsequent observations were allocated to their DTG group regardless of further changes to their ART regimen. Further, participants switching to DTG‐based regimens from a non‐suppressed state remained in the non‐DTG group, because DTG is a component of many second‐line ART regimens. This “intention to treat” analysis allows for real‐world effects to be estimated.

The routine viral load monitoring schedule in South Africa starts at 6 months after initiating ART (4 months for sites in the Western Cape), then at 1 year and then yearly thereafter. We created notional measurement points at these dates for all participants. For each notional measurement date, we used the closest viral load measurement within a 6‐month window centred around the date of the notional measurement. In a second imputation step, missing viral load measurements were imputed using multivariate imputation by chained equations. Details of the imputation process are provided in the Supplementary Material. In all the main analyses, we used a viral load threshold of 1000 HIV RNA copies/ml to determine suppression status.

When participants resume care following interruptions, their viral load monitoring schedule “resets” (i.e. their next viral load measurement is scheduled for 4 or 6 months from the date of resuming care). Treatment interruptions were determined in two ways. First, where there was a record of the next scheduled visit, participants who were more than 4 weeks late for this next visit were deemed to have interrupted ART. The 4‐week threshold is in line with the cut‐offs used by UNAIDS [25], PEPFAR [26] and the South African national ART guidelines [27]. Second, where there was no record of the next scheduled visit, we defined care interruption as a gap in contact longer than 208 days (180 days plus 28 days). Recorded contact dates included dates of clinic visits and dates of laboratory tests. We used a threshold of 180 days as sites commonly schedule 6‐monthly visits for participants deemed to be stable on treatment [28], and added 28 days for consistency with the method used in cases of there being a record of the next scheduled visit date. In both methods of determining interruptions, participants were assumed not to have interrupted ART if they had viral suppression within 2 weeks of returning to care. Participants who did not resume care were considered lost to follow‐up and censored at the date of their next scheduled visit (if available) or 180 days after their last recorded contact.

### 2.4 Statistical analyses

2.4

We performed three main analyses. All three analyses used logistic regression to model the outcome of viral non‐suppression at each measurement point. In model 1, we used the full data from 2005 to 2023 and fitted the logistic regression model using generalized estimating equations to account for patient‐level clustering. The model included the following covariates: updated age, sex, baseline CD4+ cell count, duration on ART, year of viral load measurement, cohort, DTG use (initiated or switched) and previous treatment interruptions.

Models 2 and 3 were causal analyses in which we used inverse probability weighting (IPW) to investigate the effects of DTG‐based ART on viral non‐suppression. In model 2, we estimated the effect of initiating ART on DTG‐based ART as opposed to initiating on non‐DTG‐based regimens. Only data from participants starting ART on or after 27 November 2019 were analysed because the probability of starting a first‐line DTG‐based regimen was zero before this date. Participants who later switched to DTG‐based ART from a suppressed state were censored at the date of switching. IPWs were determined using a logistic regression model to estimate the probability of starting ART on a DTG‐based regimen, where explanatory variables included only baseline covariates (sex, age, CD4+ cell count, year of starting ART and cohort). The outcome model was again fitted using generalized estimating equations. In model 3, we assessed the effect, among those already on non‐DTG‐based ART, of switching to DTG‐based regimens from a suppressed state. This required time‐updated IPWs. These weights were based on the cumulative probabilities of switching to DTG‐based ART over time, determined by fitting a Cox proportional hazards model with explanatory variables of sex, baseline age, baseline CD4+ cell count, cohort, history of previous interruptions, and most recent duration of viral suppression. Participants were not excluded if they started ART prior to 27 November 2019; however, only measurements subsequent to this date were analysed. Since the generalized estimating equations package in Stata does not currently support time‐varying weights, we instead used a standard logistic regression model, adjusting for patient‐level clustering using robust standard errors.

We performed several sensitivity analyses. First, all three of the main analyses were repeated using a lower viral suppression threshold of 400 HIV RNA copies/ml. Second, to assess the potential bias introduced by imputing baseline CD4+ cell counts (a known predictor of viral suppression), all three main analyses were repeated after excluding participants with missing baseline CD4+ cell count measurements.

Imputation was performed using R (version 4.4.1). Data preparation and regression analyses were performed using Stata (version 17).

### 2.5 Ethical considerations

2.5

The IeDEA‐SA Collaboration has ethics approval from the Human Research Ethics Committees of the University of Cape Town and the University of Bern. The seven sites each have local institutional ethics approval to contribute data to the IeDEA‐SA Collaboration. For most sites, the requirement for informed consent has been waived since only anonymized data are contributed; however, consent was obtained if required by the respective local research ethics committees.

## 3 RESULTS

We included data from 380,720 participants. Table 1 shows the baseline characteristics of the entire study population and the subgroups analysed in models 2 and 3. Overall, the median age at first ART initiation was 35.0 years (interquartile range [IQR] 28.9−42.3), and the majority of the cohort was female (64.7%). The median baseline CD4+ cell count was 236 cells/mm^3^ (IQR 125–374). The total person‐years of observation was 2, 090, 912, with a median follow‐up time of 4.9 years (IQR 2.1−8.2) per person, inclusive of periods of treatment interruption.

**Table 1:** Characteristics of participants from 2005 to 2023 in the IeDEA Southern Africa Collaboration

		All participants (analysed in model 1)	Participants analysed in model 2^a^	Participants analysed in model 3^b^
**Variable**		*N* (%) or median (IQR)	*N* (%) or median (IQR)	*N* (%) or median (IQR)
Number of participants		380, 720	46, 099 (12.1%)^c^	203, 982 (53.6%)^c^
Age at first ART initiation (years)		35.0 (28.9−42.3)	34.9 (28.2−42.5)	35.0 (29.1−42.3)
Sex	Female	246, 443 (64.7%)	29, 463 (63.9%)	134, 800 (66.1%)
	Male	134, 277 (35.3%)	16, 636 (36.1%)	69, 182 (33.9%)
Baseline CD4+ count (cells/µl)^d^		236 (125−374)	311 (163−493)	267 (150−413)
Dolutegravir use	Started on DTG	26, 993 (7.1%)	26, 993 (58.6%)	N/A
	Switched to DTG	53, 203 (14.0%)	N/A	53, 203 (26.1%)
Year of first ART initiation	2005−2007	24, 655 (6.5%)	N/A	5279 (2.6%)
	2008–2010	49, 879 (13.1%)	N/A	16, 834 (8.3%)
	2011–2013	84, 071 (22.1%)	N/A	41, 801 (20.5%)
	2014–2016	97, 074 (25.5%)	N/A	60, 127 (29.5%)
	2017–2019	79, 946 (21.0%)	1303 (2.8%)	61, 673 (30.2%)
	2020–2021	29, 608 (7.8%)	29, 390 (63.8%)	14, 692 (7.2%)
	2022–2023	15, 487 (4.1%)	15, 406 (33.4%)	3576 (1.8%)
Cohort	Aid for AIDS	148, 300 (39.0%)	16, 741 (36.3%)	83, 179 (40.8%)
	Gugulethu	14, 004 (3.7%)	393 (0.9%)	5304 (2.6%)
	Hlabisa	64, 307 (16.9%)	9306 (20.2%)	36, 998 (18.1%)
	Khayelitsha	86, 951 (22.8%)	11, 232 (24.4%)	53, 369 (26.2%)
	Khethimpilo	32, 614 (8.6%)	8121 (17.6%)	18, 169 (8.9%)
	Themba Lethu	29, 894 (7.9%)	277 (0.6%)	6644 (3.3%)
	Tygerberg	4650 (1.2%)	29 (0.1%)	319 (0.2%)

*Note*: Column percentages are shown. Years of data contribution from each site: Aid for AIDS (2006−2023), Gugulethu (2005−2021), Hlabisa (2011−2023), Khayelitsha (2007−2022), Khethimpilo (2013−2023), Themba Lethu (2005−2022) and Tygerberg (2005−2020).

Abbreviations: ART, antiretroviral therapy; DTG, dolutegravir; IQR, interquartile range.

^a^ Participants starting ART on or after 27 November 2019.

^b^ Participants starting ART on non‐DTG‐based regimens and still on ART after 27 November 2019.

^c^ Percentage of all study participants (*N* = 380, 720).

^d^ Before missing values were imputed.

Slightly over one‐fifth of participants (21.1%) either started ART on DTG‐based regimens (7.1%) or switched to DTG‐based regimens from a virally suppressed state (14.0%). Approximately half of the participants (53.0%) did not have recorded baseline CD4+ cell count measurements. The vast majority of these participants (64.0%) were from the Aid for AIDS cohort, followed by Khayelitsha (12.5%) and Hlabisa (10.6%). There were 145, 342 participants (38.2%) who experienced treatment interruptions. The median duration of interruptions was 103 days (IQR 51–324). Thirty‐six thousand three hundred and eighty‐nine participants (9.6%) were lost to follow‐up.

There were approximately 1.7 million notionally scheduled viral load measurement dates, of which 41.0% were missing recorded values (i.e. viral load tests were not performed). As presented in Figure S1, missingness of viral load measurements was positively associated with previous ART interruptions and longer duration on ART, and negatively associated with starting ART on a DTG‐based regimen and female sex. Missingness also varied by cohort and calendar year. The overall downward trend in the missingness of viral load measurements was not disrupted during the COVID‐19 pandemic period (Figure S2).

**Figure 1:**
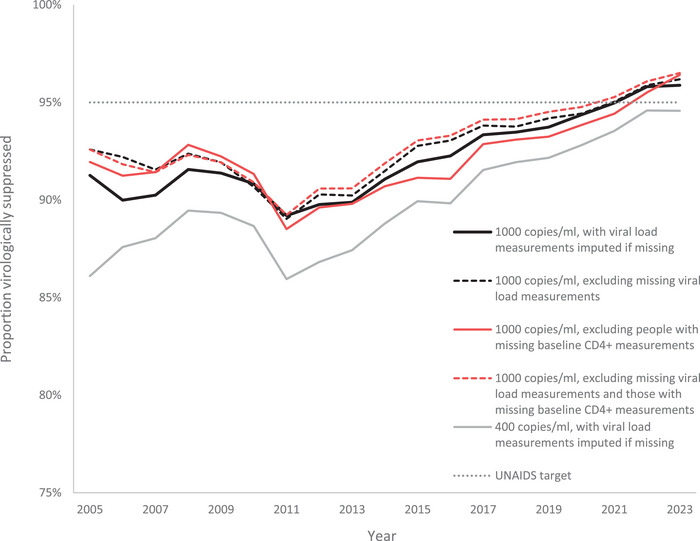
Proportion of participants on ART with viral suppression at different suppression thresholds.

The proportion of participants with viral loads less than 1000 HIV RNA copies/ml decreased from 91.3% in 2005 to 89.2% in 2011, after which there was a gradual increase to a high of 95.9% in 2023 (Figure 1). Similar trends were observed when using only recorded viral load measurements (i.e. excluding imputed measurements), when excluding those with missing baseline CD4+ cell count measurements, and when using a suppression threshold of 400 HIV RNA copies/ml.

**Figure 2:**
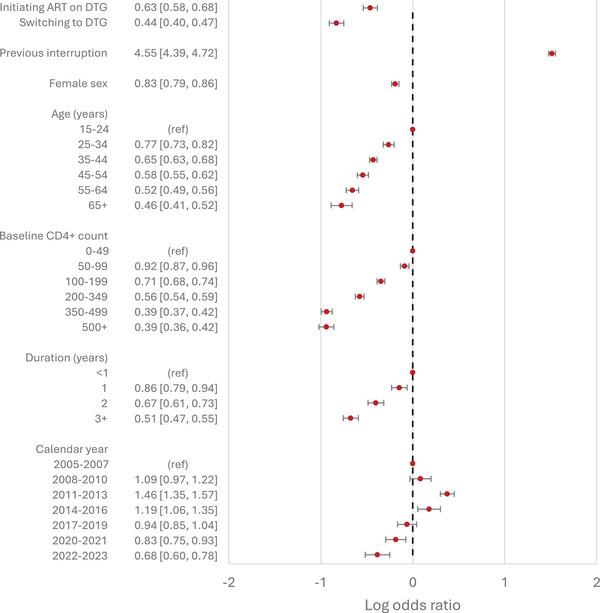
Adjusted odds ratios of viral non‐suppression from model 1, including all study participants and data from 2005 to 2023. Adjusted odds ratios are presented in the absolute scale on the left, and plotted on the log scale on the right. Results are adjusted for all variables shown, as well as clinic site. 95% Confidence intervals are indicated by horizontal bars.

**Figure 3:**
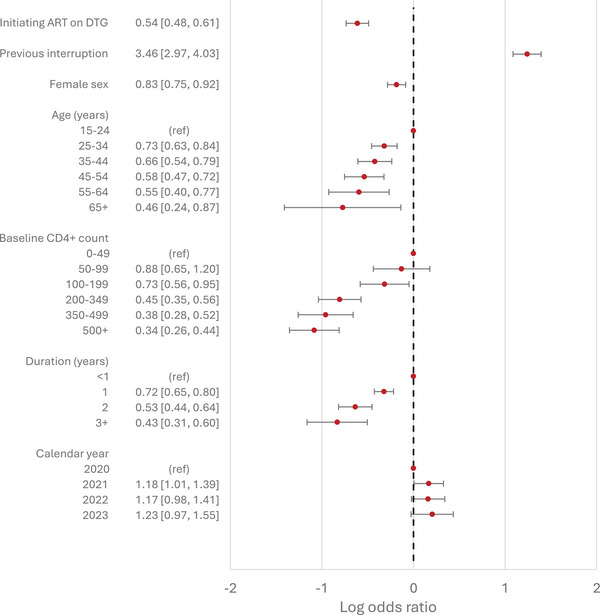
Adjusted odds ratios of viral non‐suppression from model 2, comparing those who started on DTG‐based ART with those who started non‐DTG‐based ART, using data from participants starting ART during or after November 2019. Adjusted odds ratios are presented in the absolute scale on the left, and plotted on the log scale on the right. Results are adjusted for all variables shown, as well as clinic site. 95% Confidence intervals are indicated by horizontal bars.

**Figure 4:**
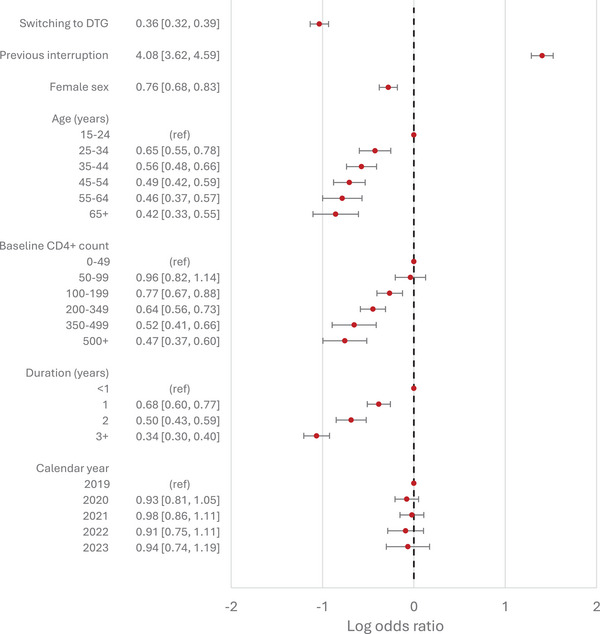
Adjusted odds ratios of viral non‐suppression from model 3, comparing those who switched to DTG‐based ART (from a suppressed state) with those who did not switch, using only data from November 2019 onwards. Adjusted odds ratios are presented in the absolute scale on the left, and plotted on the log scale on the right. Results are adjusted for all variables shown, as well as clinic site. 95% Confidence intervals are indicated by horizontal bars.

In model 1, those on DTG‐based regimens were less likely to experience viral non‐suppression, with odds ratios of 0.63 (95% confidence interval [CI]: 0.58−0.68) and 0.44 (95% CI: 0.40−0.47) for those starting ART on DTG‐based regimens and those switching to DTG‐based regimens, respectively. These odds ratios were slightly lower in the two causal analyses: 0.54 (95% CI: 0.48−0.61) for those starting ART on DTG‐based regimens (model 2) and 0.36 (95% CI: 0.32−0.39) for those switching to DTG‐based regimens (model 3). In all three main analyses, the COVID‐19 pandemic period (2020−2021) was not associated with viral suppression relative to the adjacent years. People with a history of previous treatment interruptions were more likely to experience viral non‐suppression compared to those who had not interrupted ART, with adjusted odds ratios ranging from 3.46 to 4.55. Female sex was negatively associated with viral non‐suppression, with odds ratios ranging from 0.76 to 0.83. The associations of viral non‐suppression with (updated) age, baseline CD4+ cell count and duration on ART were consistent across the three main analyses (Figures 2, 3, 4), with viral non‐suppression decreasing with increasing age, increasing duration on ART and increasing baseline CD4+ cell count.

In sensitivity analyses, the effect of DTG‐based regimens on viral non‐suppression did not vary substantially: the effect of starting ART on DTG‐based regimens compared to starting on non‐DTG‐based ART ranged from an adjusted odds ratio of 0.45 to a ratio of 0.56 (Figures S4 and S7), and the effect of switching to DTG‐based regimens compared to remaining on non‐DTG‐based ART ranged from an odds ratio of 0.34 to a ratio of 0.41 (Figures S5 and S8). The effect of previous treatment interruptions did vary significantly, with odds ratios ranging from 2.49 to 4.55: odds ratios were higher in model 1 (and related sensitivity analyses) and lower in analyses that excluded those with missing baseline CD4+ cell count measurements. Female sex was associated with lower odds of viral non‐suppression in all analyses except where those with missing baseline CD4+ cell count measurements were excluded in model 2, where there was no statistically significant effect (Figure S7). The odds ratios that were significant ranged from 0.76 to 0.83 (including results from the three main analyses). The magnitude of the effect of ART duration also varied, with the odds associated with the longest duration (3 years or more) ranging from 0.32 to 0.64. While the calendar year effects varied across analyses, the COVID‐19 pandemic period was not significantly associated with viral suppression relative to the adjacent periods in all but one of the sensitivity analyses (where those with missing baseline CD4+ cell count measurements were excluded in model 1). Full results of the sensitivity analyses are presented in the Supplementary Material (Figures S3−S8).

## 4 DISCUSSION

In this retrospective cohort study, we investigated viral non‐suppression and its predictors in South African adults on ART. We found that viral suppression has continued to improve in recent years and that DTG‐based ART has a strong effect on improved virological outcomes, both among those who initiate ART on DTG as well as those who switch to DTG while suppressed on other regimens, compared to those on non‐DTG‐based ART. A further notable finding is that those with a history of previous ART interruptions have a markedly higher risk of viral non‐suppression after returning to care.

These results are consistent with findings from previous studies. Our findings related to the protective effect of DTG are consistent with results from a 2020 meta‐analysis [29] of randomized controlled trials investigating viral suppression among those initiating ART on DTG‐based regimens. To our knowledge, there have been no randomized controlled trials comparing viral suppression among people switching to DTG‐based triple‐drug ART with those remaining on non‐DTG‐based ART regimens. While there have been several observational studies investigating these groups, almost all have included participants who were not suppressed at the time of transitioning to DTG‐based regimens [30, 31, 32, 33, 34]. These results are not comparable because those who are unsuppressed when transitioning are more likely to remain unsuppressed during the course of their follow‐up period [34]. One study in South Africa [35] that only included individuals with viral suppression at the time of transitioning reported an adjusted risk ratio of 1.01 (95% CI: 1.00−1.02) for viral suppression (as opposed to non‐suppression). While an exact conversion to the corresponding adjusted odds ratio is not possible, an approximation based on the reported proportions with viral suppression yields an odds ratio of 0.86. Several factors further complicate a direct comparison: the study only assessed viral suppression during the first year post‐transition; a lower threshold of 50 HIV RNA copies/ml was used to determine viral suppression; and approximately 15% of individuals were excluded due to missing viral load data. The missingness of viral load measurements is particularly relevant. In both our study and that of Pillay et al. [13], viral load missingness was associated with several predictors of viral suppression. Our findings, therefore, provide valuable evidence supporting the strategy of transitioning people to DTG‐based regimens from other first‐line ART.

Viral non‐suppression was not associated with the COVID‐19 pandemic period (2020–2021). This is consistent with findings from a study using specimen‐level data from the South African National Health Laboratory Service (NHLS) [36] as well as studies in other countries [37, 38, 39, 40, 41]. It also highlights the robustness of the national ART programme. Several reasons have been proposed for this resilience, including programme adaptations (such as multi‐month dispensing [20] and expansion of the Central Chronic Medicine Dispensing and Distribution programme [42]) as well as proactivity on the part of PLHIV (a study in KwaZulu‐Natal [15] found a 23% increase in ART collection visits in the month prior to the first lockdown period, after adjusting for seasonality). Additionally, the alcohol bans instituted during the lockdown periods may have exerted a compensatory effect, given the strong association between alcohol use and poor ART adherence [43].

Our estimates of the effect of previous ART interruptions on the odds of viral non‐suppression, which ranged from 2.49 to 4.55, are also consistent with previous research [44, 45, 46, 47, 48, 49, 50]. This increased risk of viral non‐suppression after resuming ART, combined with other adverse outcomes such as slower CD4+ cell count increases [2, 51, 52, 53], faster progression to AIDS [54, 55, 56, 57] and increased mortality [2, 58], indicates a need for further interventions designed to retain people on ART. This is of growing importance given that the cumulative risk of interruptions—already 38.2% in our study population—is expected to increase as people remain on ART for increasingly longer durations.

This study has three notable strengths. First, the large sample size, with over 380, 720 participants contributing more than 2, 090, 912 person‐years of observation, yielded precise estimates. Second, the extended duration of follow‐up, with participants observed for a median of 4.9 years and data spanning from 2005 to 2023, enabled us to evaluate long‐term outcomes that have not been examined in previous studies. Third, to address confounding and selection bias, we used IPW with time‐updated weights (part of a broader class of g‐methods) that allowed us to estimate the causal effects of DTG‐based ART with greater validity.

There are, however, several limitations. First, although we adjusted for a wide range of measured confounders, the use of observational data means that residual confounding is still possible. Unmeasured variables include treatment adherence and socio‐economic status, both of which may be associated with predictor variables and virological outcomes. Second, given the high proportion of missing viral load data, we needed to impute viral load measurements. This may introduce bias if viral load measurements were not missing at random, conditional on the measured confounders included in this study. However, our results were consistent across sensitivity analyses, including those that excluded participants with missing baseline CD4+ cell count measurements. Nonetheless, the imputation approach may limit generalizability in settings with missing data patterns that differ from those in our study. Last, although our definitions for ART interruption reflect national guidelines and account for differentiated models of care, they may misclassify people who transfer clinics silently or those without a record of their next scheduled visit date.

## 5 CONCLUSIONS

This study provides updated and robust estimates of viral suppression among adults receiving ART in South Africa, drawing on a large dataset and applying causal inference methods suited to longitudinal observational data. We found that DTG‐based regimens, whether started at ART initiation or started from a suppressed state, are associated with substantially improved virological outcomes. These findings add to the growing body of evidence demonstrating the effectiveness of DTG‐based ART. Our results also emphasize the large negative impact of previous ART interruptions. Taken together, these findings reinforce the importance of continuing DTG rollout and the need for interventions addressing barriers to retention in care.

## COMPETING INTERESTS

The authors declare that they have no competing interests.

## AUTHOR CONTRIBUTIONS

LFJ and HM conceptualized the study. HM, LFJ, RK, M‐AD, RdW and PN contributed to the development of the methodology. JE, GM, HWP, MPF, CO, GF and FT were responsible for data curation. HM and LFJ analysed the data and prepared the original draft. All authors reviewed and approved the final manuscript.

## FUNDING

Research reported in this publication was supported by the U.S. National Institutes of Health's National Institute of Allergy and Infectious Diseases (NIAID), the Eunice Kennedy Shriver National Institute of Child Health and Human Development (NICHD), the National Cancer Institute (NCI), the National Institute on Drug Abuse (NIDA), the National Heart, Lung, and Blood Institute (NHLBI), the National Institute on Alcohol Abuse and Alcoholism (NIAAA), the National Institute of Diabetes and Digestive and Kidney Diseases (NIDDK) and the Fogarty International Center (FIC) under Award Number U01AI069924. Haroon Moolla received training in research that was supported by the Fogarty International Center of the National Institutes of Health and the Eunice Kennedy Shriver National Institute of Child Health and Human Development (NICHD) under award number D43 TW010559.

## DISCLAIMER

This publication is subject to the NIH Public Access Policy. Through acceptance of this federal funding, NIH has been given a right to make this manuscript publicly available in PubMed Central upon the official date of publication. The content is solely the responsibility of the authors and does not necessarily represent the official views of the National Institutes of Health.

## Supporting information




**Supporting Information file 1**: Supplementary material
Microsoft Word document. This file contains additional detail on the methodology for imputing missing data, analyses of missing viral load measurements and results from sensitivity analyses.

## Data Availability

The data that support the findings of this study are not publicly available due to privacy and ethical restrictions. Data were obtained from the International Epidemiology Databases to Evaluate AIDS‐Southern Africa (IeDEA‐SA). For inquiries about the data, readers can contact IeDEA‐SA through the online form available at https://www.iedea‐sa.org/contact‐us/.
