# Supplementary material for: Estimates and predictors of HIV viral non‐suppression in South African adults on antiretroviral treatment
Source: J Int AIDS Soc. 2026 Jan 19;29(1):e70076. doi: 10.1002/jia2.70076 (PMC12816880; doi:10.1002/jia2.70076)
Supplement: Supplementary file 1 — Supporting Information file 1: Supplementary material Microsoft Word document. This file contains additional detail on the methodology for imputing missing data, analyses of missing viral load measurements and results from sensitivity analyses. [file JIA2-29-e70076-s001.pdf]

Estimates and Predictors of HIV Viral Non-suppression in South African Adults on Antiretroviral Treatment

Supplementary material

Section A: Methodology and code for imputing missing viral load measurements

1. Motivation and methodology

For longitudinal data, setting the data in "wide" format allows the imputation model to include previous and subsequent observations as predictors, thereby accounting for the autocorrelated structure of repeated measurements[1]. The imputation model differs for the first observation in that it does not include any previous observations, and similarly the model for the final observation does not include any subsequent observations. Huque et al[2] examined the case where all participants have the same period of observation (i.e. starting and ending at the same fixed times), with repeated measurements also occurring at the same time points for all participants. In this context, they compared several imputation techniques and found that multiple imputation by chained equations (MICE) performed as well as more complex techniques that attempt to account for covariance structures in the data.

However, when participants have varying starting times and durations of follow-up it is challenging to specify different imputation models for the first and last observations. Furthermore, the presence of time-varying explanatory variables very quickly leads to the problem of overfitting and collinearity[3] (in our case the use of dolutegravir, presence of interruptions, and current age will each need to have an additional column at every time point).

An alternative approach is to impute the data in long format, where time-varying explanatory variables only have one column. Here the imputation model accounts for the autocorrelated structure of repeated measurements by including the previous and next measurements as distinct variables (i.e. a column is created for each). Missing values across the three variables can then be imputed using the "Just Another Variable" (JAV) approach. The first issue that arises with this approach is that the imputed values for the previous and next measurements will almost surely be inconsistent with those of the actual (primary) measurements. Nonetheless, several studies have found that JAV performs favourably for interaction and non-linear effects compared to many other techniques.[4–7] The second problem is that imputation in the long format cannot account for clustering at the level of individual participants – the inclusion of participant identifiers naturally introduces too many additional effects. This problem may be insignificant if the data indicate that individual-level clustering is negligible relative to other sources of variation. This can easily be found by fitting mixed effects regression models.

An alternative imputation technique that has been used to account for derived variables (e.g. body mass index) and interaction terms is passive imputation.[8] In this approach imputation can be performed in the long format, with previous and next measurements as additional columns. However, instead of directly imputing these variables, they are passively updated after each iteration of the imputation algorithm, thereby maintaining their consistency between previous, current, and next viral load measurements. The challenge here is that for each participant's first and last observations there are no previous and next measurements. Imputation packages cannot proceed with incomplete predictor variables.

In this study we propose the following method to overcome this issue. First, create the following variables:

1. $Previous\_VL$ and $Next\_VL$, which are based on the observed viral load measurements. For each participant's first measurement date there is no "previous viral load". Similarly,

for each participant's final measurement there is no "next viral load". For now fill in an arbitrary value, c, for these "missing" elements.

2. Dummy variables, I_{First} and I_{Last}, indicating each participant's first and last observations, respectively.
3. Two interaction terms, $Previous\_VL_{-1} = Previous\_VL \times (1 - I_{First})$, and $Next\_VL_{-n} = Next\_VL \times (1 - I_{Last})$.

Consider the simplified scenario where viral loads are predicted only by their immediately preceding value, together with another independent variable X. To remove the effect of each participant's first "previous viral load" when imputing the missing viral loads, we define the regression model as:

$$VL = \beta_0 + \beta_{F1}.I_{First} + \beta_{F2}.Previous\_VL_{-1} + \boldsymbol{\beta}.\boldsymbol{X} + \varepsilon$$

where $\boldsymbol{X}$ is a vector of other covariates and ε is the random error term.

The imputed viral load measurements are now given by the following equations:

$VL^* = \beta_0 + \beta_{F1} + \boldsymbol{\beta}.\boldsymbol{X} + \varepsilon$ for participants' first measurements, and

$VL^* = \beta_0 + \beta_{F2}.Previous\_VL + \boldsymbol{\beta}.\boldsymbol{X} + \varepsilon$ for subsequent viral load measurements

By setting up the imputation model using the above variables and regression equation, $Previous\_VL$ plays no role in determining the first viral load measurement. There is an additional constant effect of β_1, which may be useful to capture average differences between the first and subsequent viral loads. One important such difference is the proportion of people with resistant strains of HIV – those starting ART are more likely to have contracted the resistant strain, whereas those already on ART may contract the strain or develop resistance *de novo*.

A similar logic is used to structure the imputation model to ensure that $Next\_VL$ plays no role in determining each participant's final viral load measurement. The final model for imputing missing viral loads is therefore:

$VL^* = \beta_0 + \beta_{F1} + \beta_{L2}.Next\_VL + \boldsymbol{\beta}.\boldsymbol{X} + \varepsilon$ for the first measurement,

$VL^* = \beta_0 + \beta_{L1} + \beta_{F2}.Previous\_VL + \boldsymbol{\beta}.\boldsymbol{X} + \varepsilon$ for the last measurement,

$VL^* = \beta_0 + \beta_{F2}.Previous\_VL + \beta_{L2}.Next\_VL + \boldsymbol{\beta}.\boldsymbol{X} + \varepsilon$ for other measurements.

2. R Code snippet for imputing missing viral load measurements

For the below code, the data are arranged in long format, with one row for each visit date. The data are sorted by participant, and within each person visits are sorted from earliest to latest. The previous and next viral loads (previous_vl and next_vl) have been determined and filled in, where available, as additional columns. As described above, each person's first previous_vl and last next_vl are given values of zero. This further ensures that imputation of viral loads does not use data from other participants.

```
# Between iterations, R stores arrays/lists of imputed values, not the complete
# data. The arrays have varying sizes/lengths: variables with no missing values
# have arrays of length 0, whereas a variable with n missing values has an array
# of length n.

# Indices of which VL, previous_vl and next_vl measurements are missing:
missing_VL_indexes <- which(is.na(data$VL))
missing_previous_vl_indexes <- which(is.na(data$previous_vl))
missing_next_vl_indexes <- which(is.na(data$next_vl))

# Adjusting the indices of missing previous and next viral loads to match
# the corresponding VLs (i.e. one visit before and after, respectively)
missing_previous_vl_indexes <- missing_previous_vl_indexes - 1
missing_next_vl_indexes <- missing_next_vl_indexes + 1

# **Indicators** for which VLs will be used to fill in the missing previous and
# next viral loads
temp_prev_vl <- matrix(NA, nrow=1, ncol=length(data$VL))
temp_prev_vl[missing_previous_vl_indexes] <- 1
temp_next_vl <- matrix(NA, nrow=1, ncol=length(data$VL))
temp_next_vl[missing_next_vl_indexes] <- 1
# **Indices** to use in the arrays of imputed VLs
whiches_prev <- which(!is.na(temp_prev_vl))
indexes_prev <- match(whiches_prev, missing_VL_indexes)
whiches_next <- which(!is.na(temp_next_vl))
indexes_next <- match(whiches_next, missing_VL_indexes)

# Set the imputation method for viral loads
meth <- imp$method
meth["VL"] <- "pmm"

# Set the post-processing rules
postP <- imp$post
postP["previous_vl"] <- "imp$previous_vl[[i]] <- imp$VL[[i]][indexes_prev]"
postP["next_vl"] <- "imp$next_vl[[i]] <- imp$VL[[i]][indexes_next]"

# Set the predictor matrix
predictor_matrix <- matrix(0, nrow=ncol(data), ncol=ncol(data))
rownames(predictor_matrix) <- colnames(data)
colnames(predictor_matrix) <- colnames(data)
predictor_matrix["VL", c("next_vl", "previous_vl", "I_first", "I_last", …all other
variables…)] <- 1
# For previous and next viral loads, to ensure that R doesn't ignore rows with
# missing values, these will be imputed with a simple regression model that only
# includes VL, after which the post-processing will adjust the imputed values:
predictor_matrix["previous_vl", "VL"] <- 1
predictor_matrix["next_vl", "VL"] <- 1

# Proceed with imputation
imp <- mice(data, method = meth, m = imputations, predictorMatrix =
predictor_matrix, post = postP, maxit = iterations, seed = 51423)
```

References

1. Kalaycioglu O, Copas A, King M, Omar RZ. A Comparison of Multiple-Imputation Methods for Handling Missing Data in Repeated Measurements Observational Studies. Journal of the Royal Statistical Society Series A: Statistics in Society. 2016 Jun 1;179(3):683–706.

2. Huque MH, Moreno-Betancur M, Quartagno M, Simpson JA, Carlin JB, Lee KJ. Multiple imputation methods for handling incomplete longitudinal and clustered data where the target analysis is a linear mixed effects model. Biometrical Journal. 2020;62(2):444–66.

3. Welch C, Bartlett J, Petersen I. Application of multiple imputation using the two-fold fully conditional specification algorithm in longitudinal clinical data. Stata J. 2014 Apr 1;14(2):418–31.

4. Von Hippel PT. How to Impute Interactions, Squares, and Other Transformed Variables. Sociological Methodology. 2009;39(1):265–91.

5. Seaman SR, Bartlett JW, White IR. Multiple imputation of missing covariates with non-linear effects and interactions: an evaluation of statistical methods. BMC Med Res Methodol. 2012 Apr 10;12(1):46.

6. Kim S, Belin TR, Sugar CA. Multiple imputation with non-additively related variables: Joint-modeling and approximations. Stat Methods Med Res. 2018 Jun 1;27(6):1683–94.

7. De Silva AP, Moreno-Betancur M, De Livera AM, Lee KJ, Simpson JA. A comparison of multiple imputation methods for handling missing values in longitudinal data in the presence of a time-varying covariate with a non-linear association with time: a simulation study. BMC Med Res Methodol. 2017 Jul 25;17(1):114.

8. White IR, Royston P, Wood AM. Multiple imputation using chained equations: Issues and guidance for practice. Stat Med. 2011 Feb 20;30(4):377–99.

Section B: Analyses of missing viral load measurements

Figure S1 shows the results from a logistic regression model fitted to estimate the probability of a viral load measurement being missing. Robust standard errors were used to account for patient-level clustering.

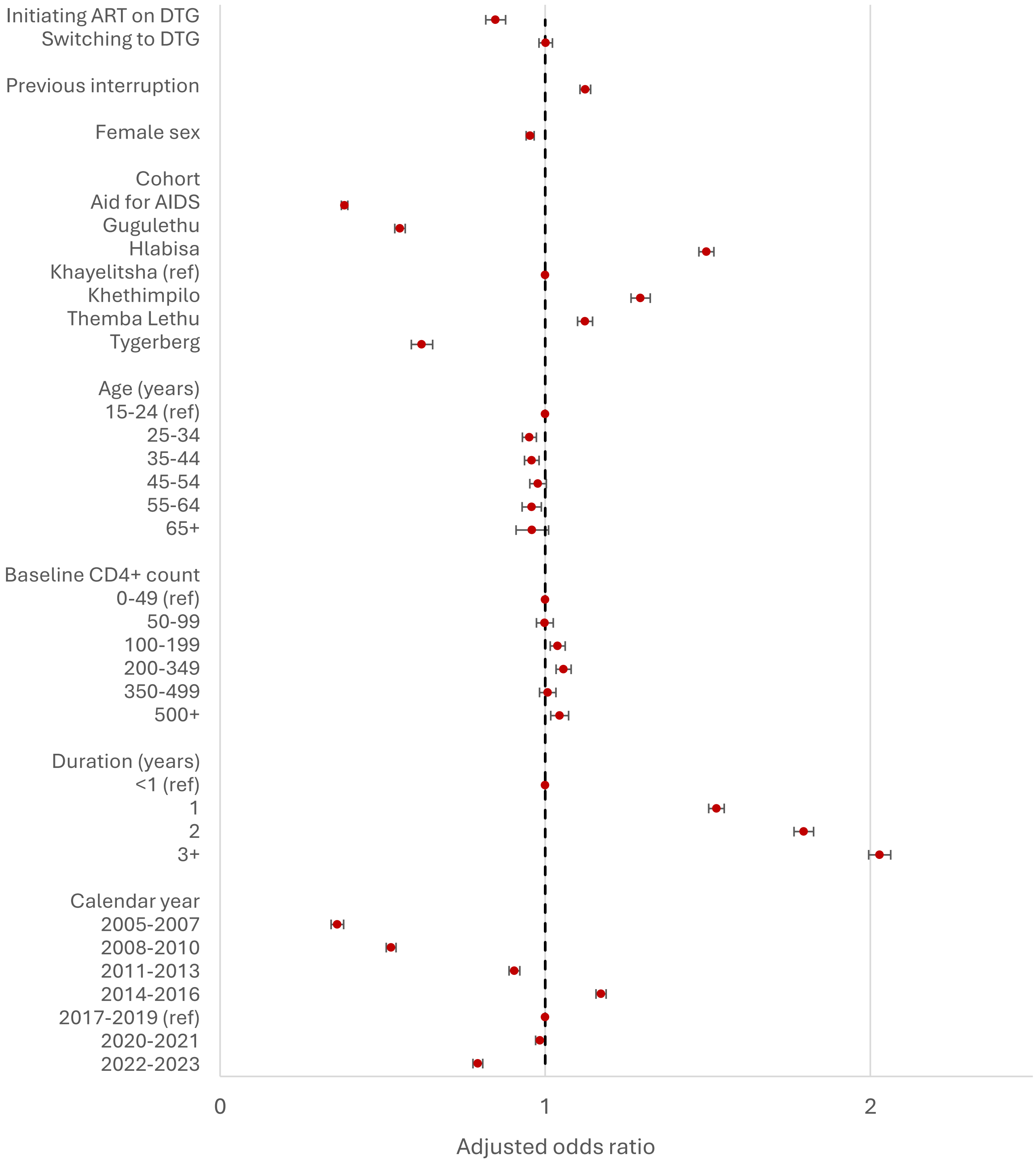

Figure S1. Adjusted odds ratios for variables associated with missing viral load measurements. 95% Confidence intervals are indicated by horizontal bars.

Figure S2 shows the proportion of participants without viral load measurements over time. For each year, the numerator is equal to the number of participants without a recorded viral load measurement that year, while the denominator is equal to the number of participants with at least one recorded visit that year. Note that cohorts only started contributing data from the first year in which viral load testing was performed for at least 40% of participants in that cohort.

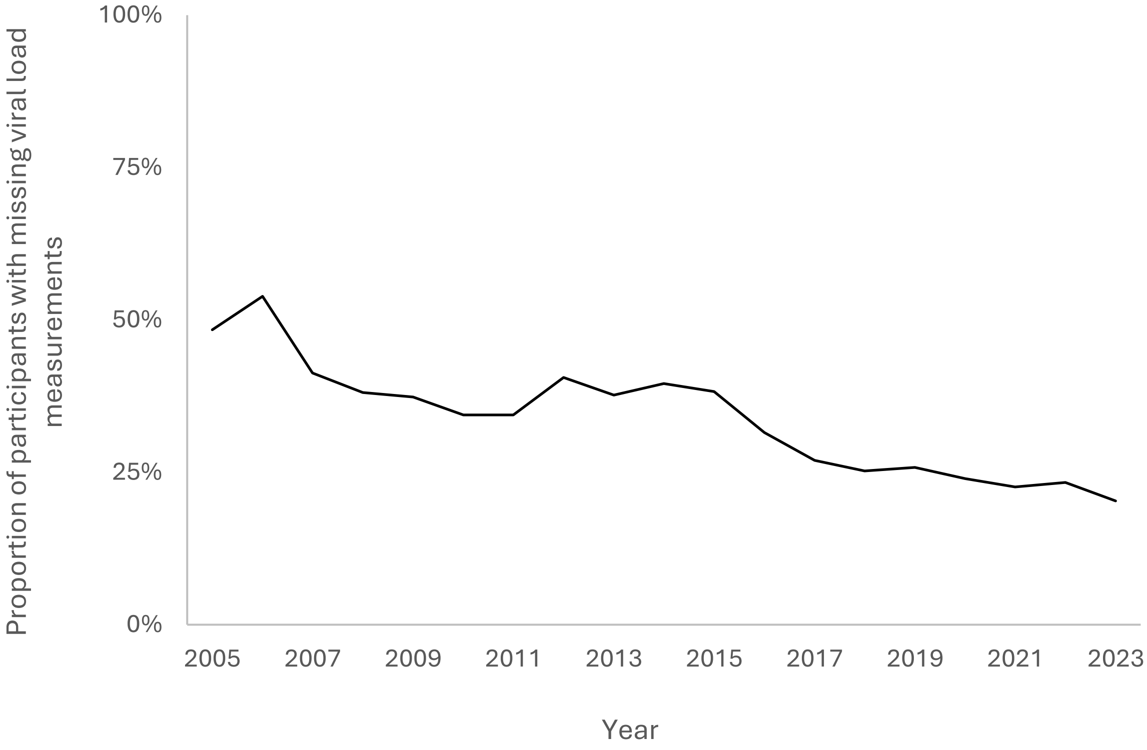

Figure S2. Proportion of participants with missing viral load measurements over time. Each cohort only contributed data from the first year in which viral load testing was performed for at least 40% of participants in that cohort.

Section C: Results from sensitivity analyses

1. Analyses using a viral suppression threshold of 400 copies/ml.

Initiating ART on DTG	0.74 [0.68, 0.80]
Switching to DTG	0.48 [0.44, 0.53]
Previous interruption	4.13 [4.02, 4.25]
Female sex	0.83 [0.80, 0.86]
Age (years)	
15-24	(ref)
25-34	0.77 [0.72, 0.83]
35-44	0.67 [0.63, 0.72]
45-54	0.62 [0.57, 0.66]
55-64	0.56 [0.51, 0.61]
65+	0.50 [0.44, 0.56]
Baseline CD4+ count	
0-49	(ref)
50-99	0.95 [0.91, 1.00]
100-199	0.76 [0.73, 0.79]
200-349	0.61 [0.59, 0.63]
350-499	0.45 [0.42, 0.48]
500+	0.43 [0.40, 0.46]
Duration (years)	
<1	(ref)
1	0.84 [0.80, 0.88]
2	0.67 [0.62, 0.72]
3+	0.53 [0.49, 0.56]
Calendar year	
2005-2007	(ref)
2008-2010	1.12 [1.02, 1.23]
2011-2013	1.57 [1.40, 1.76]
2014-2016	1.29 [1.17, 1.42]
2017-2019	0.99 [0.89, 1.10]
2020-2021	0.89 [0.80, 0.99]
2022-2023	0.72 [0.62, 0.84]

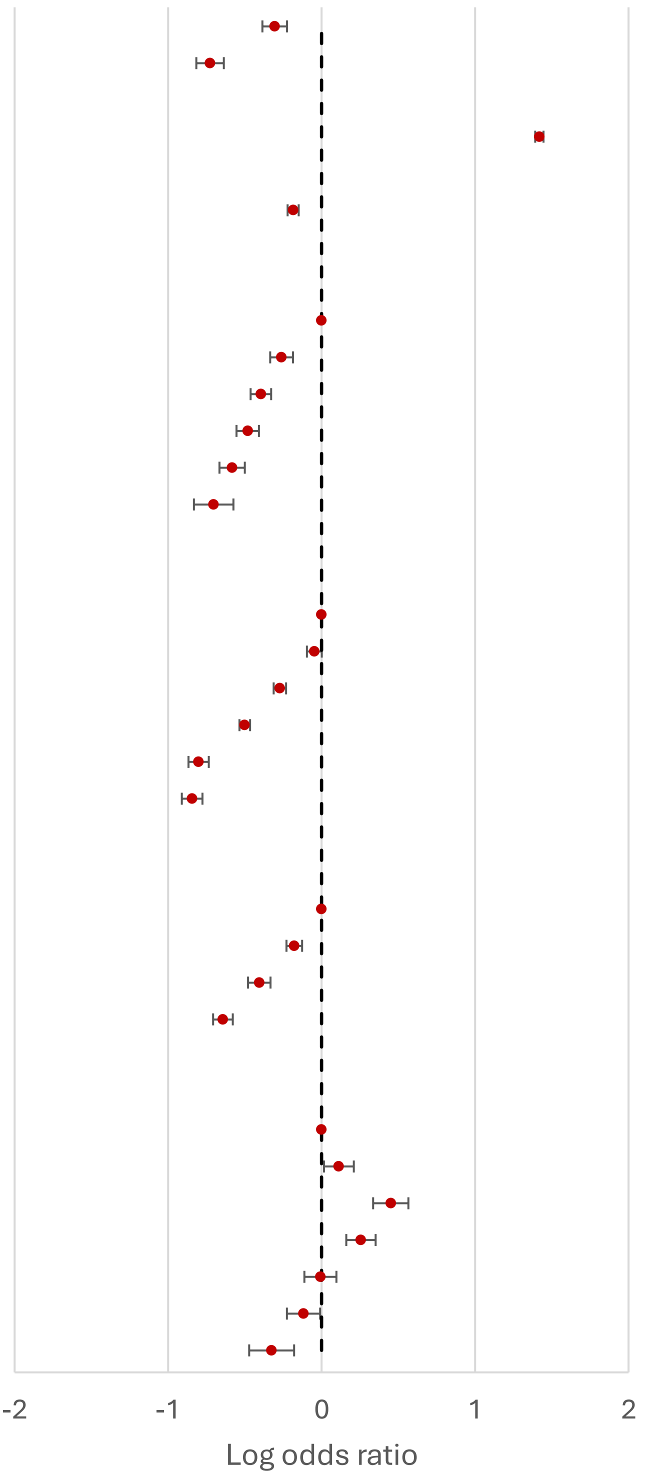

Figure S3. Adjusted odds of virological failure from model 1, using a viral suppression threshold of 400 copies/ml. Adjusted odds ratios are presented in the absolute scale on the left, and plotted on the log scale on the right. Results are adjusted for all variables shown, as well as clinic site. 95% Confidence intervals are indicated by horizontal bars.

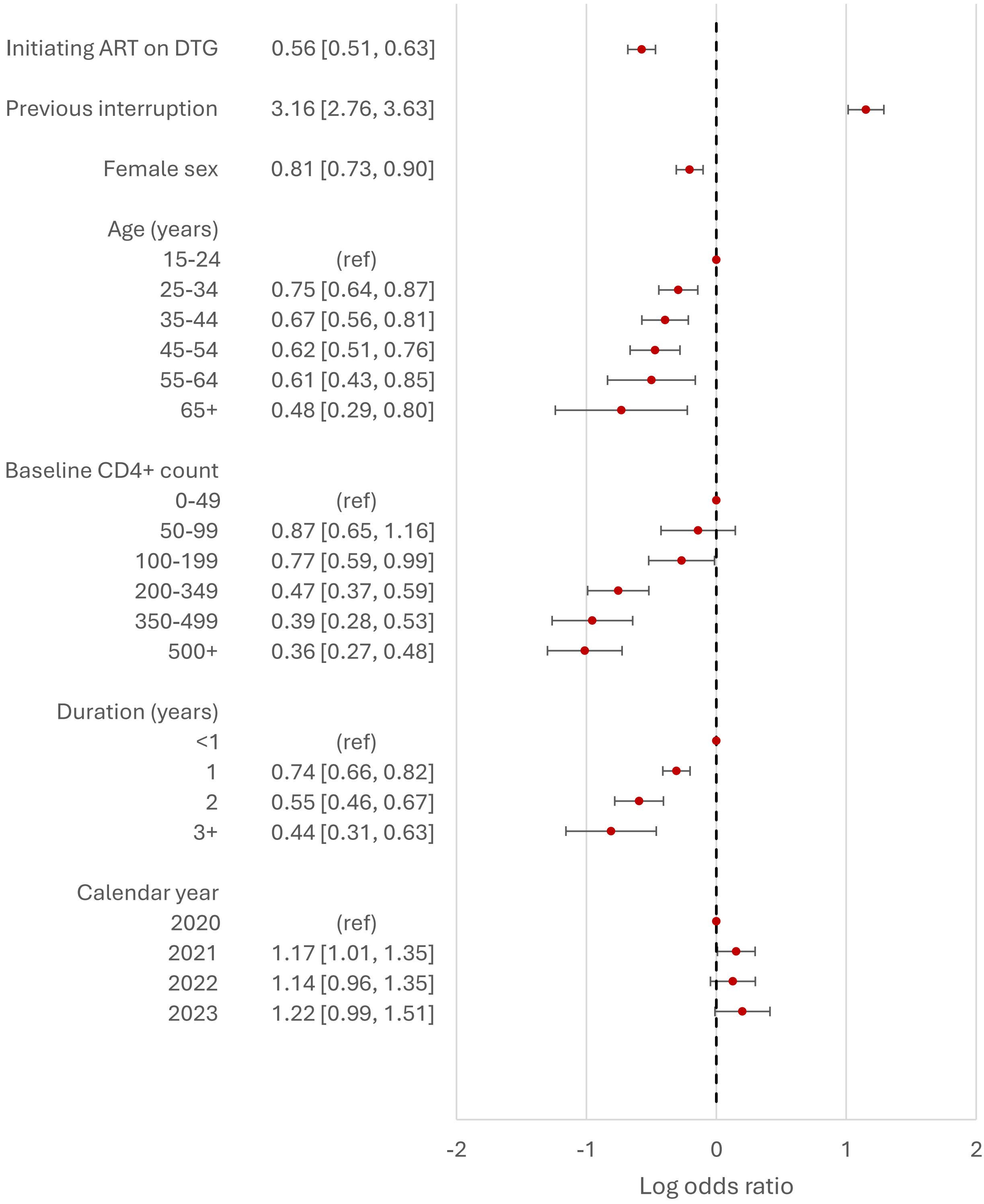

Figure S4. Adjusted odds of virological failure from model 2 using a viral suppression threshold of 400 copies/ml, comparing those who started on DTG-based ART with those who started non-DTG-based ART. Adjusted odds ratios are presented in the absolute scale on the left, and plotted on the log scale on the right. Results are adjusted for all variables shown, as well as clinic site. 95% Confidence intervals are indicated by horizontal bars.

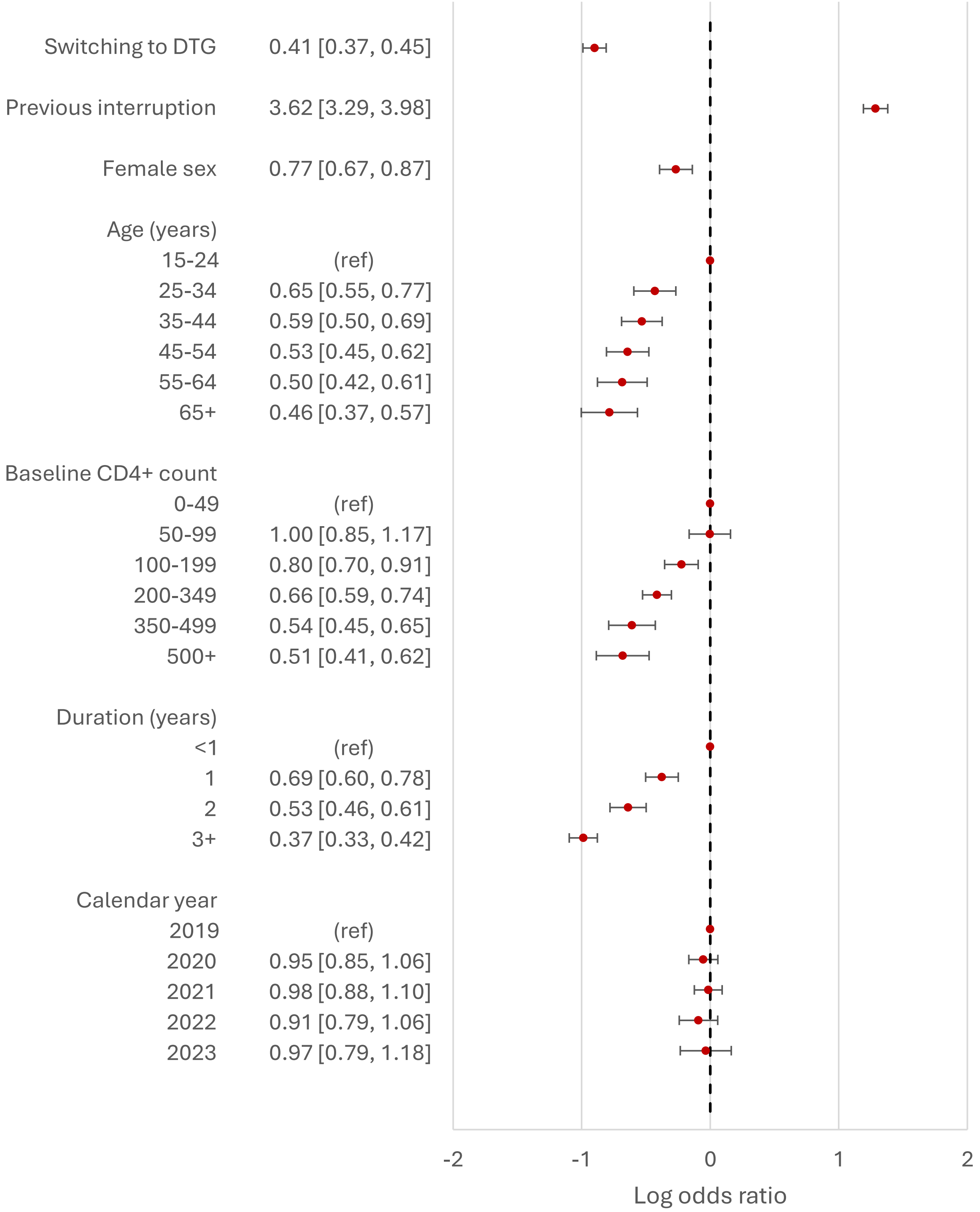

Figure S5. Adjusted odds of virological failure from model 3, using a viral suppression threshold of 400 copies/ml, comparing those who switched to DTG-based ART (from a suppressed state) with those who did not switch. Adjusted odds ratios are presented in the absolute scale on the left, and plotted on the log scale on the right. Results are adjusted for all variables shown, as well as clinic site. 95% Confidence intervals are indicated by horizontal bars.

2. Analyses excluding those with missing baseline CD4+ cell count measurements, and using a viral suppression threshold of 1000 copies/ml.

Initiating ART on DTG	0.72 [0.64, 0.81]
Switching to DTG	0.45 [0.39, 0.51]
Previous interruption	3.10 [2.99, 3.21]
Female sex	0.82 [0.80, 0.84]
Age (years)	
15-24	(ref)
25-34	0.80 [0.75, 0.85]
35-44	0.66 [0.61, 0.71]
45-54	0.59 [0.55, 0.63]
55-64	0.54 [0.49, 0.59]
65+	0.51 [0.44, 0.59]
Baseline CD4+ count	
0-49	(ref)
50-99	0.87 [0.83, 0.92]
100-199	0.70 [0.67, 0.73]
200-349	0.48 [0.45, 0.50]
350-499	0.36 [0.34, 0.38]
500+	0.30 [0.28, 0.33]
Duration (years)	
<1	(ref)
1	0.95 [0.92, 0.99]
2	0.79 [0.76, 0.82]
3+	0.64 [0.61, 0.67]
Calendar year	
2005-2007	(ref)
2008-2010	1.11 [1.02, 1.20]
2011-2013	1.74 [1.61, 1.89]
2014-2016	1.55 [1.43, 1.70]
2017-2019	1.20 [1.10, 1.32]
2020-2021	1.14 [1.03, 1.26]
2022-2023	0.89 [0.80, 0.99]

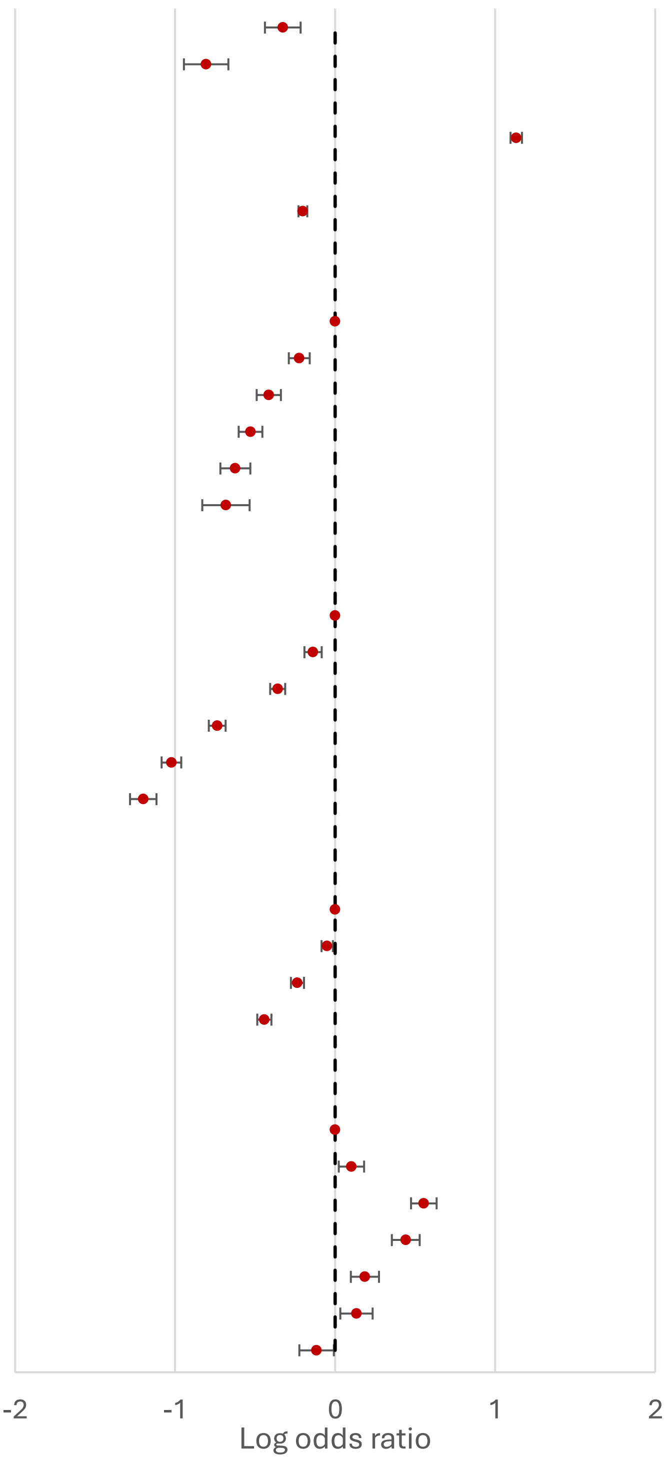

Figure S6. Adjusted odds of virological failure from model 1, excluding those with missing baseline CD4+ cell count measurements. Adjusted odds ratios are presented in the absolute scale on the left, and plotted on the log scale on the right. Results are adjusted for all variables shown, as well as clinic site. 95% Confidence intervals are indicated by horizontal bars.

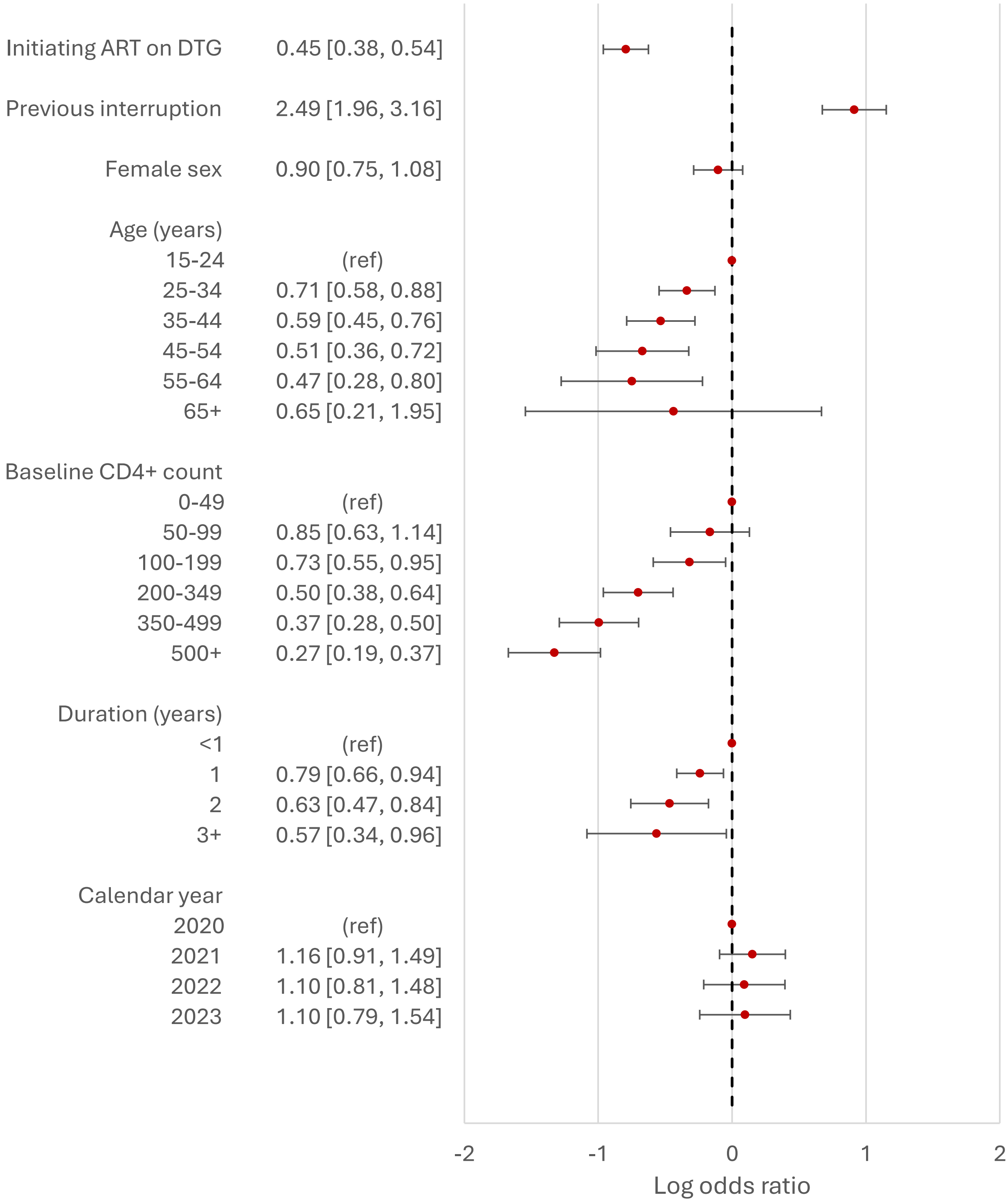

Figure S7. Adjusted odds of virological failure from model 2, excluding those with missing baseline CD4+ cell count measurements, comparing those who started on DTG-based ART with those who started non-DTG-based ART. Adjusted odds ratios are presented in the absolute scale on the left, and plotted on the log scale on the right. Results are adjusted for all variables shown, as well as clinic site. 95% Confidence intervals are indicated by horizontal bars.

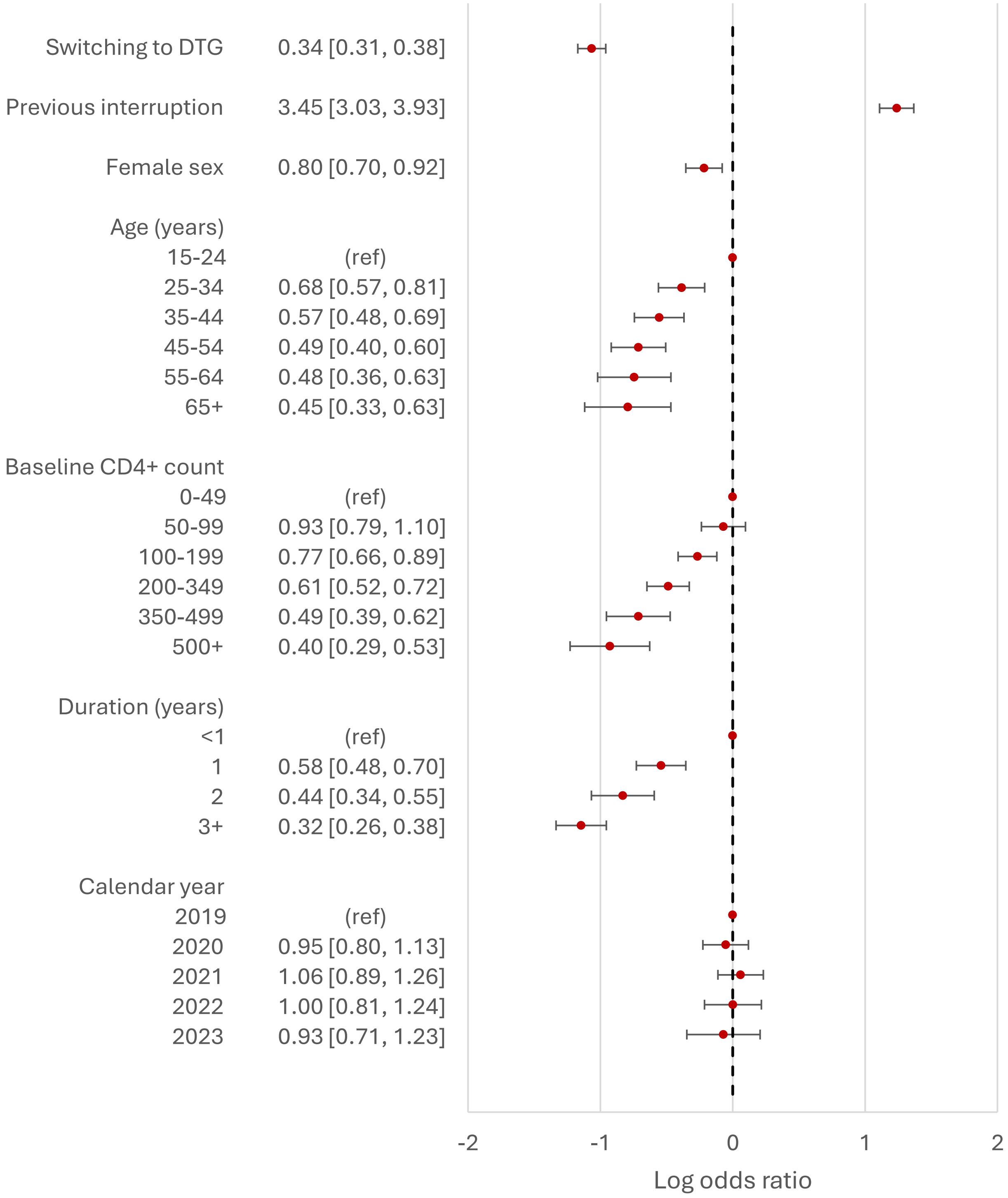

Figure S8. Adjusted odds of virological failure from model 3, excluding those with missing baseline CD4+ cell count measurements, comparing those who switched to DTG-based ART (from a suppressed state) with those who did not switch. Adjusted odds ratios are presented in the absolute scale on the left, and plotted on the log scale on the right. Results are adjusted for all variables shown, as well as clinic site. 95% Confidence intervals are indicated by horizontal bars.
